# microRNA-193a-3p is specifically down-regulated and acts as a tumor suppressor in *BRAF*-mutated colorectal cancer

**DOI:** 10.1186/s12885-017-3739-x

**Published:** 2017-11-07

**Authors:** Hidekazu Takahashi, Masanobu Takahashi, Shinobu Ohnuma, Michiaki Unno, Yuki Yoshino, Kota Ouchi, Shin Takahashi, Yasuhide Yamada, Hideki Shimodaira, Chikashi Ishioka

**Affiliations:** 1 Department of Clinical Oncology, Institute of Development, Aging and Cancer, Tohoku University, 4-1 Seiryo-machi, Aoba-ku, Sendai, Miyagi 980-8575 Japan; 2 Department of Medical Oncology, Tohoku University Hospital, Sendai, Miyagi 980-8574 Japan; 3 Department of Surgery, Tohoku University Graduate School of Medicine, Sendai, Miyagi 980-8574 Japan; 4 Department of Gastrointestinal Medical Oncology, National Cancer Center Hospital, Tokyo, 104-0045 Japan

**Keywords:** Colorectal cancer, BRAF, miRNA, miR-193a-3p, Anti-EGFR therapy

## Abstract

**Background:**

The aim of this study was to identify miRNAs specifically dysregulated in *BRAF*-mutated colorectal cancer, which could lead to a better understanding of the molecular mechanisms underlying oncogenesis of this malignant subtype of colorectal cancer.

**Methods:**

Candidate dysregulated miRNAs were selected in genome-wide miRNA expression array analysis using a screening set composed of 15 *BRAF*-mutated and 15 non-*KRAS/BRAF*-mutated colorectal cancers. The miRNA expressions were validated in another set of patients. The functional roles of the miRNAs were analyzed by cell growth and invasion assays. The association between miRNA expression status and the clinical outcome of patients treated with various chemotherapies was analyzed.

**Results:**

Within the top five of the miRNAs screened, we validated miRNA-31 (miR-31) and miR-135b as up-regulated, while miR-193a-3p was down-regulated in *BRAF*-mutated cancer. Moreover, miR-193a-3p inhibited cell growth, and invasion of colorectal cancer cells. Low miR-193a-3p expression was associated with shorter progression-free survival in patients who received anti-EGFR therapy.

**Conclusions:**

Our results disclose a novel tumor suppressive role of miR-193a-3p in colorectal cancer. These results could lead to novel therapeutic strategies for colorectal cancer, particularly in *BRAF*-mutated colorectal cancer.

**Electronic supplementary material:**

The online version of this article (10.1186/s12885-017-3739-x) contains supplementary material, which is available to authorized users.

## Background

RAF family kinases, including BRAF and RAF1, function downstream of RAS as critical regulators of the MEK-ERK MAP kinase signaling pathway [1]. This RAS-RAF-MEK-ERK cascade is a key pathway, which contributes to human oncogenesis controlling the cell cycle, proliferation, differentiation, angiogenesis, apoptosis, migration, and metastasis [2–4]. Since the first identification of the *BRAF* gene mutation in human cancer [5], accumulating evidence has shown that a considerable proportion of various human malignancies, such as malignant melanoma (~50%) and other solid cancers including thyroid cancer (~60%), colorectal cancer (~10%), and lung cancer (~6%) carry the activated *BRAF* mutations, which leads to constitutive activation of downstream RAF, MEK and ERK [1].

In colorectal cancer, mutations of *BRAF* predominantly occur in codon 600, particularly leading to p.V600E mutation [6]. Recently, several lines of evidence suggest that colorectal cancer containing a *BRAF* p.V600E mutation possesses more malignant potential when compared with other genotypes of colorectal cancer, including the *KRAS*/*BRAF*-wild-type tumor and the *KRAS*-mutated tumor. In resectable colon cancer patients treated with adjuvant drug therapy, the *BRAF* mutation has been associated with poor survival compared to wild-type *BRAF* [7, 8]. A similar tendency has been observed in studies of metastatic or recurrent colorectal cancer, where *BRAF* mutations are associated with worse overall survival (OS) [9, 10]. In addition to the negative prognostic impact, a *BRAF* mutation is likely to have a predictive value for resistance to anti-EGFR therapies, such as monoclonal antibodies cetuximab and panitumumab [11, 12]. However, its mutational status as a predictive marker has not been well established in clinical use.

Earlier studies have also shown that *BRAF* mutations substantially overlap with other genetic and epigenetic subtypes of colorectal cancer, such as the microsatellite instability (MSI) phenotype characterized by change in the length of simple nucleotide repeats resulting from mismatch repair deficiency, and the CpG island methylator phenotype (CIMP) characterized by widespread hypermethylation of CpG islands [13, 14]. These molecular subgroups, particularly *BRAF*-mutant tumors and CIMP-positive tumors, are associated with the serrated pathway that plays a role in colorectal tumorigenesis, which is distinct from the well-characterized chromosomal instability pathway [15]. Patients with colorectal cancer containing a *BRAF* mutation or those with CIMP-positive tend to be older, smokers, women, right-sided, and have a higher-grade histology [16, 17]. In light of the aforementioned evidence and other findings that patients with *BRAF*-mutant and/or CIMP-positive colorectal cancer (particularly without MSI or impaired mismatch repair) exhibit worse clinical outcomes [7, 10, 18], this subtype should be regarded as distinct from other molecular subtypes of colorectal cancer and should be treated using different strategies. The reason why this molecular subtype exhibits more malignant potential still remains to be elucidated, and promising molecular targets for therapy against this subtype remain to be identified.

microRNAs (miRNAs) are a class of small non-coding RNA, which exert their tumor suppressive and/or oncogenic functions primarily by binding to the 3′-untranslated region of the mRNA of target genes. The binding of miRNA to each mRNA leads to the inhibition of translation and/or enhanced degradation of the corresponding transcripts. Alteration of miRNA expression has been implicated in oncogenesis from early to late stages in various human cancers including colorectal cancer [19, 20]. There are an increasing number of studies including ours that have analyzed the functional role of miRNAs in colorectal cancer, such as miRNA-21 (miR-21), miR-31, miR-34b/c, miR-135b, miR-137, miR-143, miR-145, miR-148a, miR-200, and miR-203 [21–29]. Moreover, a few reports have focused on the relationship between *BRAF* mutations and some miRNA alterations in other cancers, although their miRNA expression profiles were not similar [30–32]. However, whether the *BRAF*-mutant-specific miRNAs can contribute to oncogenesis of this more malignant subtype of colorectal cancer, remains unclear. If the miRNA-dependent mechanisms underlying the oncogenesis of *BRAF*-mutant colorectal cancer are identified, this could lead to discovering novel molecular targets for therapy to improve the outcome of patients with colorectal cancer and even other cancers harboring *BRAF* mutations.

In this study, we aimed to identify miRNAs that are specifically dysregulated in *BRAF*-mutant colorectal cancer using a genome-wide miRNA expression analysis, and to clarify whether these miRNAs play a role in colorectal tumorigenesis as an oncogene or a tumor-suppressor through functional assays using colorectal cancer cell lines. Moreover, we investigated whether the expression of the candidate BRAF-related miRNA, miR-193a-3p, was associated with the clinical outcome of patients with metastatic colorectal cancer treated with anti-EGFR therapy.

## Methods

### Patients

A total of 314 patients with colorectal cancer, comprising 255 patients who underwent drug therapy including cytotoxic agents and anti-EGFR antibody, and/or surgery in the Tohoku University Hospital (TUH) between 2004 and 2013, and 59 patients who received anti-EGFR antibody in the National Cancer Center Hospital (NCCH) between 2003 and 2012, were recruited in this study. The clinical information regarding clinical characteristics of patients and tumors, OS, progression-free survival (PFS) after initiations of drug therapies, and response rate (RR), was retrospectively analyzed through reviews of clinical records. As listed in Additional file 1: Table S1, clinical characteristics of patients within the TUH cohort are associated with earlier clinical stage and proximal location compared to those within the NCCH cohort.

### DNA and RNA extraction

DNA was extracted from a 5 μm- or 10 μm-thick formalin-fixed paraffin-embedded (FFPE) tissue of each patient with colorectal cancer through the use of QIAmp DNA FFPE tissue kit (Qiagen, Valencia, CA, USA). Total RNA including miRNA fraction was extracted from the FFPE tissue of each colorectal cancer by using the Ambion RecoverAll Total Nucleic Acid Isolation Kit (Life Technologies Corporation, Carlsbad, CA, USA). Total RNA was also extracted from normal adjacent colonic mucosa of 11 patients with colorectal cancer from the cohort.

### KRAS and BRAF sequencing

The mutational status of codon 12 and 13 of *KRAS* gene and the codon 600 of *BRAF* gene were analyzed by direct DNA sequencing through the use of CEQ2000EX automated DNA sequencer (Beckman Coulter, Brea, CA, USA). The accession number of cDNAs of *KRAS*, wild-type and p.V600E *BRAF*, were NM_033360.3, NM_004333.5 and HQ224878.1, respectively. Primers used for the amplification of fragments were 5′-accttatgtgtgacatgttc (forward) and 5′-atggtcctgcaccagtaata (reverse) for *KRAS* codons 12 and 13, and 5′-ataatgcttgctctgatagg (forward) and 5′-gtaactcagcagcatctcag (reverse) for *BRAF* codon 600.

### Screening of miRNAs that are dysregulated in BRAF-mutant tumors by using miRNA microarray

The genome-wide miRNA expression levels of the 30 colorectal cancers from the screening set were analyzed by the SurePrint G3 Human miRNA Rel. 16.0 microarray (Agilent Technologies, Santa Clara, CA, USA), which covers 1222 human miRNAs, according to the manufacturer’s protocol. The microarray data were extracted using the GeneSpring ver. 12.5 (Agilent Technologies). The raw data was normalized by using the 90-percentile shift method, and the acquired data of each miRNA were compared between wild-type *KRAS/BRAF* tumors and mutant-*BRAF* tumors using Mann-Whitney U test. The microarray data has been deposited in the Gene Expression Omnibus database (accession No. GSE66548).

### Quantification of miRNA expression levels by quantitative real-time RT-PCR

The miRNA expressions of colorectal tissues and colon cancer cell lines were quantified by Taqman real-time RT-PCR (qRT-PCR) using a CFX96 real-time PCR detection system (Bio-Rad Laboratories, Hercules, CA, USA). The relative expression of each miRNA was calculated by the delta CT value method, through the use of miR-16 expression for human colon samples [27, 33] and RNU48 for colorectal cancer cell lines [34] as a normalizer. At least two independent samples were loaded as an internal control in each PCR plate for miR-193a-3p analysis for colorectal tumors, to keep consistency of measurements throughout all plates. Each sample was amplified in triplicate and the results obtained from each run were normalized according to the data of internal controls.

### Cell lines

Human colorectal cancer cell lines RKO (CRL-2577) and HCT116 (CCL-247) were purchased from the American Type Culture Collection in 2011. Human colorectal cancer cell lines DiFi, HCT8, LIM2405, and SW48 were kindly provided along with appropriate ethics rules and consents of both institutions by Dr. Mariadason in Ludwig Institute for Cancer Research, Australia. The cell lines were regularly authentificated by short tandem repeat analysis. RKO was cultured in Dulbecco’s Modified Eagle’s Medium (Sigma-Aldrich, St.Louis, MO, USA) with 10% fetal bovine serum and the other four cell lines were grown in Roswell Park Memorial Institute Medium 1640 (Sigma-Aldrich) with 10% fetal bovine serum at 37 °C.

### Pre-miR-193a-3p and anti- miR-193a-3p transfection

The cells were transfected with precursor of miR-193a-3p or precursor of negative control (PM11123 or AM17110, Applied Biosystems), or anti-miR-193a-3p or anti-negative control (AM17000 or AM17010, Applied Biosystems) at a final concentration of 33–67 nM using Lipofectamine 2000 (Life Technologies Corporation), according to the manufacturer’s protocol.

### Cell growth assay

The cells were seeded onto 96-well plates with the different number of cells (RKO, 7 × 10^3^; HCT116, 5 × 10^3^; SW48, 1.5 × 10^4^). When attached, cells were transfected with precursors of miR-193a-3p or negative control as mentioned above. The cell viability was measured after 48 h in RKO or after 72 h in HCT116 and SW48 using the Cell Counting Kit-8 (Dojindo Laboratories, Kumamoto, Japan), according to the manufacturer’s protocol. Each experiment was performed in quadruplicate and data were obtained from three or more independent experiments.

### Invasion assay

Invasion activities of RKO and HCT116 cells were analyzed using Boyden chambers with 8-mm pore membranes coated with matrigel (BD Biosciences, San Jose, CA, USA) following the standard protocol. In six-well plates, 3 × 10^5^ of RKO cells and 1 × 10^5^ of HCT116 cells were transfected with precursors of miR-193a-3p or negative control. After 24 h, the transfected cells (3 × 10^5^ of the RKO cells and 1.5 × 10^5^ of the HCT116 cells) were re-suspended in 500 μl of serum-free medium in the upper wells. Medium containing 10% fetal bovine serum was then added into the bottom wells. After incubation for 48 h for RKO or 24 h for HCT116, the invaded cells were stained with 0.2% crystal violet solutions. The number of cells was counted from four representative fields of each membrane, and the results were obtained from three independent experiments.

#### Quantification of gene expression levels by qRT–PCR

Total RNA was extracted using the RNeasy Mini Kit (Qiagen), and cDNA was synthesized from mRNA using the iScript advanced cDNA Synthesis Kit (Bio-Rad Laboratories) following the manufacturer’s protocol. To quantify gene expression levels of *ZEB1*, *ZEB2*, *SNAI1*, and *SNAI2*, qRT–PCR was performed on the CFX96 real-time PCR detection system using SYBR® Green PCR Master Mix (Applied Biosystems) following the manufacturer’s protocol. Each gene expression level was normalized to an expression level of *GAPDH*. The primers used were as follows: *ZEB1* forward; 5-ttcaaacccatagtggttgct, *ZEB1* reverse; 5-tgggagataccaaaccaactg, *ZEB2* forward; 5-caagaggcgcaaacaagc, *ZEB2* reverse; 5-ggttggcaataccgtcatcc, *SNAI1* forward; 5-gctgcaggactctaatccaga, *SNAI1* reverse; 5-gctgcaggactctaatccaga, *SNAI2* forward; 5-tggttgcttcaaggacacat, *SNAI2* reverse; 5-gttgcagtgagggcaagaa, *GAPDH forward*; 5-acccagaagactgtggatgg, *GAPDH* reverse; 5-cagtgagcttcccgttcag.

### BRAF transfection

Human wild-type *BRAF* cDNA was prepared by PCR using the primers (forward; 5′-gtggaattctgcagatataagatggcggcgctgagcggtgg, reverse; 5′-gccactgtgctggatcctttgttgctactctcctgaactctctcactc), which cover full lengths of the coding region of the gene, from a human cDNA library. The amplified fragments were cloned into the pcDNA 3.1(+) vector (Life Technologies) using the In-Fusion HD Cloning Kit (Takara Bio, Shiga, Japan). Human mutant *BRAF* cDNA containing p.V600E mutation was constructed by site-directed mutagenesis using the wild-type *BRAF* expression vector as a template. The insert fragments containing wild-type or mutant *BRAF* was sequenced by ABI Prism 3130 (Life Technologies), to confirm that the fragments has no sequence variation in the coding region of *BRAF* other than p.V600E.

### Western blot analysis

Western blot analysis was performed following a standard protocol [35]. Anti-BRAF rabbit monoclonal antibody (#9433, Cell Signaling Technology, Danvers, MA, USA), anti-p-BRAF (Ser445) rabbit monoclonal antibody (#2696, Cell Signaling Technology), anti-p-MEK1/2 (Ser217/221) rabbit polyclonal antibody (#9121, Cell Signaling Technology), anti-p-ERK1/2 (Ser217/221) rabbit monoclonal antibody (#4094, Cell Signaling Technology), and anti-α-tubulin mouse monoclonal antibody (Sigma-Aldrich) were used as primary antibodies for detection of the specific proteins.

### Statistical analysis

Statistical analyses were performed with JMP Pro ver. 11.0 (SAS Institute, Cary, NC, USA). The differences between two groups were analyzed by chi-square test, Fisher’s exact test, Student’s *t* test or Mann-Whitney U-test, depending on each parameter. Correlation analysis was performed by using Spearmann’s rank correlation method. Kaplan-Meier analysis was conducted to estimate distributions of OS, or PFS after the beginning of the first-line chemotherapy or after anti-EGFR therapies, and a log-rank test was utilized to analyze the statistical difference in the survival. Each difference was regarded as statistically significant when *P* < 0.05.

### Ethics statement

This study was performed in accordance with the Declaration of Helsinki and was approved by the Ethical Committee of TUH and NCCH. A written informed consent was obtained from all patients.

## Results

### Screening of BRAF-mutant specific miRNAs

**Fig. 1:**
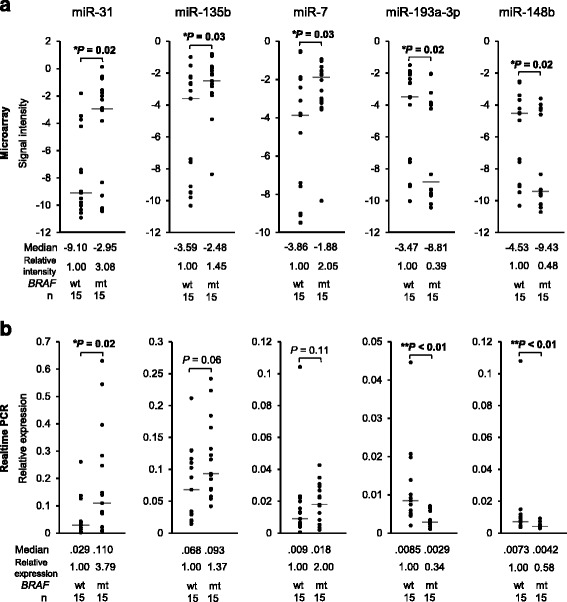
Screening of candidate miRNAs, which were significantly altered in *BRAF*-mutant colorectal cancers (*n* = 15) compared to *KRAS/BRAF*-wild-type colorectal cancers (n = 15), using a genome-wide miRNA expression analysis. **a** The top five dysregulated miRNAs from the miRNA microarray analysis. **b** The results of the microarray analysis were technically validated using Taqman real-time RT-PCR. Mann–Whitney U test was used to analyze statistical differences

We first analyzed the mutational status of *KRAS* and *BRAF* in colorectal cancers from our cohort of patients. The sequencing analyses identified *KRAS* mutations in 95 tumors and *BRAF* mutations in 21 tumors within both the TUH and NCCH cohort (Additional file 1: Table S1). Two tumors with concurrent mutations within both *KRAS* and *BRAF* genes were excluded from any further analysis. To screen the miRNAs that are dysregulated in *BRAF*-mutant tumors, we then divided 64 tumor samples from the entire cohort into two sets as follows: a screening set (15 *KRAS*/*BRAF*-wild-type tumors and 15 *BRAF*-mutant tumors from the TUH and NCCH cohort) and a validation set (30 *KRAS*/*BRAF*-wild type tumors and four *BRAF*-mutant tumors from the TUH cohort) (Table 1). Using the screening set, we found nine up-regulated miRNAs (median, > 1.5-fold) and 13 down-regulated miRNAs (median, <−1.5-fold), through the global miRNA expression analysis (Table 2). We selected the top three up-regulated miRNAs (miR-31, miR-135b, and miR-7) and the bottom two down-regulated miRNAs (miR-193a-3p and miR-148b), which had a median fold change of either >4-fold or <−4-fold respectively, as the candidates of specifically dysregulated miRNAs in *BRAF*-mutant tumors (Table 2 and Fig. 1a). Before proceeding to the next step using the validation set, we validated the expression of the five miRNAs in the screening set using qRT-PCR. The qRT-PCR results exhibited the same trend as the microarray results, except the difference in the expression level of two miRNAs, miR-135b and miR-7, where comparison between *KRAS*/*BRAF*-wild-type tumors and *BRAF*-mutant tumors became not significant (Fig. 1b). We further confirmed that the results were well correlated between the microarray and the qRT-PCR analysis by comparing the signal intensity obtained by the microarray analysis and the CT values of miR-193a-3p or miR-16 obtained by qRT-PCR in each sample (Additional file 2: Figure S1), indicating the reliability of the screening results obtained by the microarray analysis.

**Table 1:** Clinical characteristics between a screening and a validation set of patients

Characteristic	Screening set (n = 30)					Validation set (n = 34)				
	*KRAS/BRAF* wt(n = 15)		*BRAF* mt(n = 15)			*KRAS/BRAF* wt(n = 30)		*BRAF* mt(n = 4)		
	n	%	n	%	*P*	n	%	n	%	*P*
Age										
Median	66		68		0.80^a^	67		77		0.054^a^
Range	39–82		27–87			50–92		69–87		
Gender										
Men	11	73	11	73	1.00^b^	17	57	2	50	1.00^b^
Women	4	27	4	27		13	43	2	50	
Stage										
I	0	0	0	0	0.55^c^	1	3.3	0	0	0.62^c^
II	3	20	4	27		0	0	0	0	
III	6	40	3	20		24	80	4	100	
IV	6	40	7	46		5	16	0	0	
Unknown	0	0	1	7		0	0	0	0	
Histology										
Pap	0	0	0	0	0.32^c^	0	0	0	0	0.86^c^
Tub	13	86	8	53		26	87	4	100	
Por	1	7	2	13		1	3	0	0	
Muc	1	7	3	20		1	3	0	0	
Sig	0	0	1	7		0	0	0	0	
Unknown	0	0	1	7		2	7	0	0	
Location										
Proximal	3	20	8	53	0.06^b^	9	30	3	75	0.12^b^
Distal	12	80	6	40		21	70	1	25	
Unknown	0	0	1	7		0	0	0	0	

^a^Mann-Whitney U test

^b^Fisher’s exact test

^c^chi-square tests were used for the comparison of categorical variables between *KRAS/BRAF*-wild-type and *BRAF*-mutant cancers among the screening or the validation set

**Table 2:** Up-regulated and down-regulated miRNAs of *BRAF*-mutant colorectal cancer samples screened using a miRNA microarray analysis

Up-regulated miRNA	Fold change	Down-regulated miRNA	Fold change
hsa-miR-31	10.842	hsa-miR-193a-3p	−4.852
hsa-miR-135b	6.652	hsa-miR-148b	−4.704
hsa-miR-7	5.589	hsa-miR-3687	−3.611
hsa-miR-330-3p	3.481	hsa-miR-365	−3.459
hsa-miR-146a	3.335	hsa-miR-133a	−2.867
hsa-miR-138-2	2.214	hsa-miR-30e	−2.826
hsa-miR-222	1.835	hsa-miR-769-5p	−2.182
hsa-miR-501-5p	1.705	hsa-miR-214	−2.177
hsa-miR-142-3p	1.696	hsa-miR-224	−2.115
		hsa-miR-335	−2.028
		hsa-miR-1290	−1.720
		hsa-miR-663	−1.684
		hsa-miR-3656	−1.578

Up-regulated and down-regulated miRNAs that exhibited *P* < 0.05 and median’s fold change > |1.5| determined by Mann-Whitney U test were presented

### Validation of candidate miRNAs that are specifically dysregulated in BRAF-mutant tumors

**Fig. 2:**
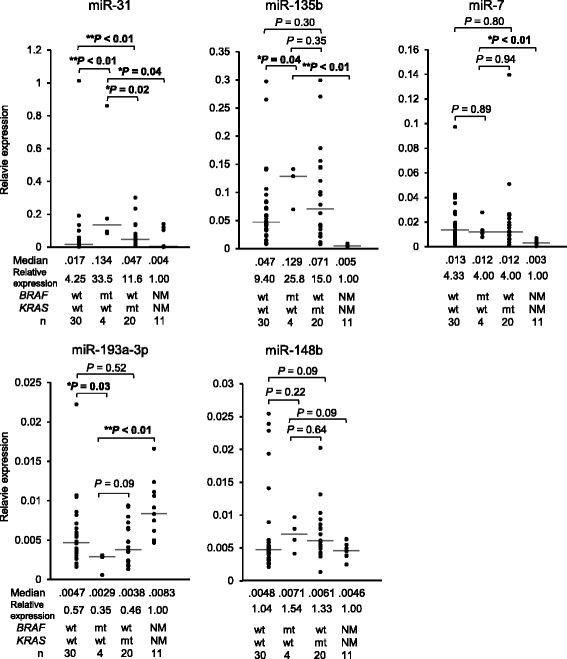
Validation of the miRNAs dysregulated in *BRAF*-mutant tumors, in another set of patients with colorectal cancer. The expression levels of the five miRNAs were validated in a different set including *KRAS/BRAF*-wild-type (*n* = 30) and *BRAF*-mutant colorectal cancers (*n* = 4). *KRAS*-mutant cancers (*n* = 20) and adjacent normal mucosa (*n* = 11) were additionally analyzed for the five miRNA expression. Mann-Whitney U test was used to analyze statistical differences

To validate the results obtained from the screening analysis, we next performed the qRT-PCR in another set of samples, the validation set. This validation analysis successfully confirmed that miR-31 and miR-135b were significantly up-regulated, and miR-193a-3p was significantly down-regulated in *BRAF*-mutant colorectal cancers compared to *KRAS*/*BRAF*-wild-type cancers (Fig. 2). In contrast, the results of miR-7 and miR-148b were not validated in this analysis (Fig. 2). In addition, to further elucidate whether the altered expression of the candidate miRNAs occurred in a *BRAF*-dependent fashion, we added *KRAS*-mutant colorectal cancers (*n* = 20) to the qRT-PCR and compared the miRNA expressions between *BRAF*-mutant tumors and *KRAS*-mutant tumors. We found that miR-31 and miR-135b were significantly up-regulated, while, miR-193a-3p was marginally significantly down-regulated (*P* = 0.09) in *BRAF*-mutant cancers compared to *KRAS*-mutant cancers (Fig. 2). Furthermore, these three miRNAs were shown to be significantly dysregulated in *BRAF-*mutant cancers compared to normal colonic mucosa (*n* = 11; Fig. 2), which suggests that the alteration in these miRNA expressions may contribute, at least in part, to the carcinogenesis of *BRAF*-mutant tumors. Of these miRNAs, up-regulation of miR-31 and miR-135b has previously been linked to tumorigenesis of colorectal cancer [21, 25]. In contrast, little is known about the functional role of miR-193a-3p for colorectal carcinogenesis. We therefore decided to focus on miR-193a-3p for further molecular and clinical analyses, to clarify the previously unknown role of miR-193a-3p, which is involved in the tumorigenesis of human colorectal cancer.

### miR-193a-3p serves as a tumor-suppressor in colorectal cancer cell lines

**Fig. 3:**
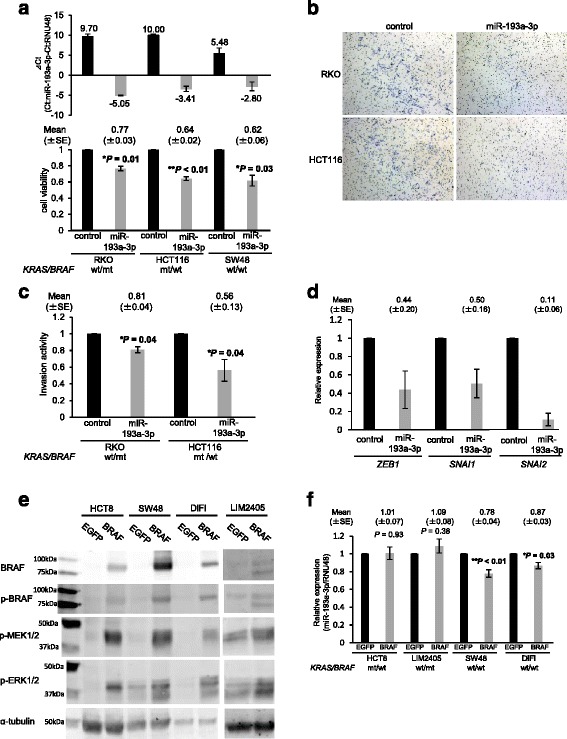
miR-193a-3p functions as a tumor-suppressor in colorectal cancer cells and its expression was decreased by overexpression of a BRAF protein. **a** Transfection of precursors of miR-193a-3p increased the expression of mature miR-193a-3p (top), and inhibited cell viability of the three cell lines irrespective of their *KRAS/BRAF* mutational status (bottom). **b, c** Transfection of the precursors of miR-193a-3p inhibited cell invasion ability in RKO and HCT116 cells. **d** miR-193a-3p overexpression reduced the mRNA levels of EMT-related genes *ZEB1*, *SNAI1,* and *SNAI2* in RKO cells. **e** The mutant BRAF (V600E) overexpression activates its downstream pathway. Western blot analysis shows that the overexpression of mutant BRAF protein caused an increase in phosphorylated levels of BRAF and its downstream MEK and ERK, in the HCT8, LIM2405, SW48 and DiFi colorectal cancer cells compared to those transfected with a control vector. **f** miR-193a-3p expression was decreased in the *KRAS/BRAF*-wild-type SW48 and DiFi cells, but not in the *KRAS*-mutant HCT8 cells and the *BRAF*-mutant LIM2405 cells 72 h after overexpression of mutant BRAF protein. The Student’s t test was used to analyze statistical differences

In light of recent evidence that miR-193a-3p may have a tumor suppressive function in cancers of other organs, such as breast and lung [36, 37], and our finding that this miRNA was down-regulated in *BRAF*-mutant colorectal cancers (Figs. 1 and 2), we hypothesized that miR-193a-3p serves as a tumor-suppressive miRNA in colorectal cancer. To address this question, we first analyzed the effect of miR-193a-3p overexpression in colorectal cancer cell lines using the cell viability and invasion assay. The cell viability assay revealed that miR-193a-3p overexpression significantly inhibited cell viability compared to overexpression of the precursors of a negative control in all three colorectal cancer cell lines analyzed (23–38%; Fig. 3a). This inhibitory effect of miR-193a-3p overexpression on cell survival did not depend on the cellular genotype of *KRAS*/*BRAF* (RKO; wild/mutant, HCT116; mutant/wild, SW48; wild/wild, respectively) [38, 39]. In addition, the invasion assay demonstrated that miR-193a-3p exerted an inhibitory effect on cellular invasion with a 20% and 50% reduction in RKO and HCT116 cells, respectively (Figs. 3b and c). Second, we analyzed the effect of miR-193a-3p inhibition in these cell lines on cell viability and invasion. Cell viability and invasion ability were not increased in these cells transfected with miR-193a-3p inhibitors (data not shown). One possible explanation might be that endogenous miR-193a-3p expression, regardless of further forced down-regulation of the miRNA, was already down-regulated in these cells, enough for affecting their viability and invasion ability. Third, in light of the observation that miR-193a-3p inhibited the invasion ability of the cancer cells, we analyzed the effect of miR-193a-3p overexpression on expressions of epithelial-mesenchymal-transition-related (EMT-related) genes such as *ZEB1*, *ZEB2*, *SNAI1*, and *SNAI2*. We found that the expression of *ZEB1*, *SNAI1*, and *SNAI2* was decreased in RKO cells transfected with the pre-miR-193a-3p compared to those transfected with a negative control (Fig. 3d), while the expression of *ZEB2* was not detected in RKO cells transfected with the negative control or those transfected with pre-miR-193a-3p. This result suggests that the inhibition of the invasion ability of cancer cells by miR-193a-3p may be induced, at least in part, through the down-regulation of EMT-related genes. These results support our surmise that miR-193a-3p functions as a tumor suppressor underlying tumor initiation and development of colorectal cancer, particularly *BRAF*-mutant tumors.

### A relationship between miR-193a-3p expression and overexpression of the BRAF protein

**Fig. 4:**
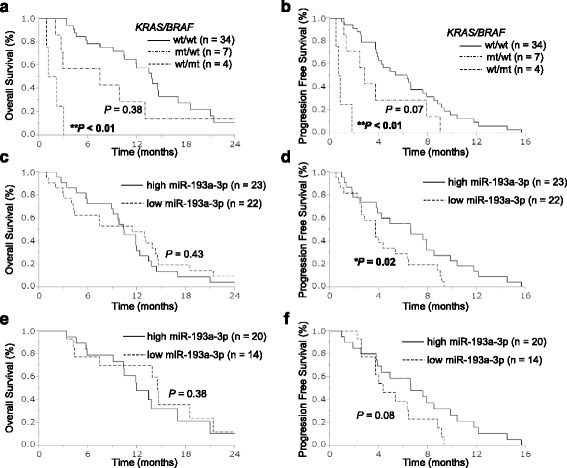
Low miR-193a-3p expression is associated with a worse survival of colorectal cancer patients treated with anti-EGFR therapy. Kaplan–Meyer curves for **a** OS from the start of anti-EGFR therapy and **b** PFS based upon the *KRAS/BRAF* mutational status (*n* = 45). Kaplan-Meyer curves for **c** OS and **d** PFS based upon miR-193a-3p expression in patients with any *KRAS/BRAF* mutational status (n = 45). Kaplan-Meyer curves for **e** OS and **f** PFS based upon miR-193a-3p expression in the *KRAS/BRAF*-wild-type group (*n* = 34)

Our screening and validation analyses identified miR-193a-3p as one possible *BRAF*-mutant specific miRNA; however, the underlying mechanisms controlling the specific down-regulation of this miRNA in *BRAF*-mutant cancers are still unclear. Earlier studies suggested that the promoter hypermethylation of miR-193a-3p may be an explanation of its reduced expression [40–42]; however, we hypothesized that BRAF or its down-stream proteins may directly affect the down-regulation of miR-193a-3p expression. To address this issue, we next examined the expression of miR-193a-3p in colorectal cancer cell lines with mutant BRAF protein overexpression. In all three cell lines transfected with a mutant-*BRAF* expression vector, we confirmed that phosphorylated levels of BRAF protein, as well as those of its downstream proteins MEK and ERK, were increased, compared to the corresponding cell lines transfected with an EGFP control vector (Fig. 3e). In this system, the expression levels of miR-193a-3p were modestly but significantly decreased in SW48 and DiFi, both *KRAS*/*BRAF*-wild-type colorectal cancer cells (Fig. 3f) [39, 43]. This modest decrease in miR-193a-3p expression levels could be due to imperfect transfection efficiency (approximately 50% EGFP positive cells in all cell lines; data not shown). In contrast, the expression of miR-193a-3p was not altered by BRAF overexpression in *KRAS*-mutant HCT8 cells and BRAF-mutant LIM2405 cells (Fig. 3d) [44], in which the downstream constituents of the RAS-RAF-MEK-ERK pathway were already constitutively active, due to the activated mutation within the upstream *KRAS*. We next tried to elucidate whether miR-193a-3p expression is affected by inhibition of the downstream constituents of the RAS-RAF-MEK-ERK pathway in a *BRAF*-mutant cell line RKO and a *KRAS*-mutant cell line HCT116. miR-193a-3p expression was not significantly affected by treatments with both a BRAF inhibitor dabrafenib and a MEK inhibitor trametinib (using two doses with 1.5 and 0.25 μM, and 3.0 and 0.5 μM, respectively) either in a *BRAF*-mutant cell line RKO or in a *KRAS*-mutant cell line at 3, 6, and 9 h treatment (Additional file 3: Figure S2). In addition, to elucidate whether the miR-193a-3p down-regulation induced by BRAF overexpression affect its target genes, we co-transfected a psiCHECK-2 vector that has a luciferase sequence with target sequences of miR-193a-3p (miCheck miRNA biosensor clone, Promega), and either the BRAF V600E overexpression vector or control pEGFP vector into *KRAS/BRAF*-wild SW48 cells. The luciferase activity was not significantly increased in cells co-transfected with pBRAF and psiCHECK-2 vector that has miR-193a-3p target sequences (Additional file 4: Figure S3). These results suggests that mutant BRAF may affect miR-193a-3p expression to some extent, but is not a single factor that contributes to dysregulation of miR-193a-3p and its target genes. However, based upon the statistically significant down-regulation of miR-193a-3p by transient overexpression of BRAF V600E as shown in Fig. 4d, the possibility exists that the mechanism underlying the miR-193a-3p down-regulation in *BRAF*-mutant colorectal cancers may be partially influenced by a direct or indirect effect of activated BRAF.

### miR-193a-3p expression status correlates with the clinical outcome of patients with colorectal cancer treated with anti-EGFR antibody therapy

Our finding that miR-193a-3p was a candidate miRNA dysregulated in a *BRAF*-dependent manner and also functioned as a tumor suppressor in colorectal cancer, prompted us to further elucidate whether its expression status was associated with the clinical outcome of our cohort of patients treated with cytotoxic chemotherapy and/or molecular-targeted drugs. We conducted a survival analysis using 99 patients from the TUH cohort, whose information on clinical outcomes such as OS, PFS for cytotoxic chemotherapy or anti-EGFR antibodies, RR, and RNA samples for the miRNA expression analysis were available. We first analyzed the OS and PFS for first-line drug therapy, and the PFS for anti-EGFR antibody therapy based upon the molecular subtypes characterized by *KRAS*/*BRAF* genotype. Consistent with earlier studies [9, 10], patients with a *BRAF*-mutant colorectal cancer had a poorer OS compared to those with a *KRAS*/*BRAF*-wild-type and *KRAS-*mutant cancer (Additional file 5: Figure S4a). In addition, as expected, *BRAF*-mutant patients showed a worse OS and PFS for anti-EGFR therapy compared to *KRAS*/*BRAF*-wild-type patients (Fig. 4a and b), whereas PFS for first-line chemotherapy of the three subtypes of patients did not differ significantly (Additional file 5: Figure S4b). Next, we categorized all colorectal tumors into a high and low miR-193a-3p expression group using the median value in all samples as a cut-off point. We found that miR-193a-3p expression status did not correlate with OS or PFS for a first-line drug therapy or OS after the initiation of anti-EGFR therapy (Additional file 5: Figure S4c, S4d and Fig. 4c). However, low expression of this miRNA was associated with a reduced PFS (HR 2.10, *P* = 0.02; Fig. 4d) and tended to be associated with a lower RR (24% vs. 42%; Additional file 1: Table S2) for anti-EGFR therapy. Since our results showed that miR-193a-3p was identified as a mutant-*BRAF*-specific miRNA, the worse outcome of colorectal cancer patients with the down-regulation of miR-193a-3p from anti-EGFR therapy may be confounded by the *BRAF*-mutation status. To avoid this possibility, we analyzed PFS for anti-EGFR therapy in our cohort of patients without *KRAS*/*BRAF* mutation (*n* = 34). In particular, we found that low miR-193a-3p expression did not associate with a poorer OS (Fig. 4e), but still tended to be associated with a shortened PFS for anti-EGFR therapy among non-*KRAS*/*BRAF*-mutant patients, although it should be noted the difference did not reach the significance level (HR 1.97, *P* = 0.08; Fig. 4f), which could be due to the small number of patients analyzed. The low miR-193a-3p expression tended to be associated with a lower RR (31% vs. 47%; Additional file 1: Table S3) as well.

Taken together, our results suggest that miR-193a-3p may not only serve as a tumor-suppressive miRNA involved in the oncogenesis of colorectal cancer, particularly *BRAF*-mutant tumors, but also may be a determinant that can affect the sensitivity to anti-EGFR therapy even in *BRAF*-wild-type colorectal cancer.

## Discussion

The purpose of this study was to identify miRNAs that were specifically dysregulated in human *BRAF*-mutant colorectal cancer, a molecular subtype of colorectal cancer with a higher malignant potential. We also sought to elucidate the functional significance of the miRNAs in colorectal cancer. We demonstrated a novel role of miR-193a-3p in colorectal cancer. First, using genome-wide miRNA expression analysis for a set of patients with colorectal cancer followed by the validation analysis for another set of patients, we identified miR-193a-3p as a down-regulated miRNA in *BRAF*-mutant colorectal cancer. Second, miR-193a-3p functioned as a tumor suppressor in a panel of colorectal cancer cell lines. Third, miR-193a-3p was partly affected by overexpression of mutant BRAF proteins. Finally, low miR-193a-3p expression status correlated with a worse clinical outcome to anti-EGFR therapy, independent of the *BRAF* mutation status. Taken together, our results indicate that the dysregulation of miR-193a-3p is involved in the tumorigenesis of colorectal cancer, particularly *BRAF*-mutant cancer, and is likely to affect drug sensitivities to anti-EGFR therapy in colorectal cancer regardless of *BRAF* mutational status. These data provide new insights into the molecular mechanisms underlying the oncogenesis of colorectal cancer.

Few studies have investigated the relationship between *BRAF* mutations and altered miRNA expression in colorectal cancer. In contrast, a handful of studies have focused on the specific alterations of miRNA expressions in *BRAF*-mutant cancer of other organs. Cahill et al. reported that 15 miRNAs were up-regulated and 23 miRNAs including miR-193a were down-regulated in *BRAF*-mutant thyroid cancer cell lines compared to normal thyroid cells [30]. Caramuta et al. reported that miR-193a-3p, miR-338 and miR-565 were under-expressed in melanomas with *BRAF* mutations compared to those without *BRAF* or *NRAS* mutations [31]. Our result of the specific down-regulation of miR-193a-3p in *BRAF*-mutant colorectal tumors is in line with these previous reports, suggesting that miR-193a-3p may be involved in oncogenesis of various malignancies with mutated *BRAF*.

More recently, Nosho et al. found that miR-31 is the most overexpressed miRNA in *BRAF*-mutant colorectal cancers. This is the first study that analyzed the association between altered miRNA expressions and *BRAF* mutations in colorectal cancer [45]. Through a global miRNA expression analysis covering 760 miRNAs, they have identified 33 dysregulated miRNAs, all of which were up-regulated in *BRAF*-mutant colorectal cancer. Our result that miR-31 was one of the most up-regulated miRNA in *BRAF*-mutant colorectal cancers is consistent with their report [45]. In addition, we found that miR-135b was also up-regulated, although it was not identified as among the 33 up-regulated miRNAs in their study. A more recent study also found miR-31 was the most up-regulated miRNA in *BRAF*-mutant tumors through screening the miRNA expression profiles of seven patients with *BRAF*-mutant tumors [46]; however, the other nine miRNAs dysregulated in their study did not include miR-7, miR-135b, miR-148b, or miR-193a-3p that were detected as dysregulated in our study. We identified miR-193a-3p as a novel down-regulated miRNA in *BRAF*-mutant colorectal cancer, which offers more information on the significance of the dysregulation of multiple miRNAs in colorectal tumorigenesis of this molecular subtype.

Our results obtained from the functional assays using a panel of colorectal cancer cell lines also support a possible role of miR-193a-3p as a tumor suppressor in colorectal cancer. To the best of our knowledge, our study is the first to demonstrate the tumor suppressive role of miR-193a-3p in colorectal cancer. The precise molecular mechanisms by which miR-193a-3p inhibits cellular oncogenic process, that is, the genes or pathways that this miRNA directly dictates in colorectal cancer, remains to be elucidated. However, recent evidence from the studies on other cancers offers some clues to this question. Uhlmann et al. demonstrated that three miRNAs, miR-124, miR-147, and miR-193a-3p, co-target EGFR-related pathway proteins, leading to an inhibition of cell-cycle progression and cell proliferation in breast cancer cell models through global miRNA screening using a high-throughput proteomic analysis [36]. miR-193a-3p has been shown to directly target *JNK1,* and overexpression of miR-193a-3p decreased the protein levels of JNK1 as well as CDK4, cyclin D1, and PIK3CA, which are all involved in the EGFR/cell cycle network pathways [36]. A more recent study by *Yu* et al. supported the role of miR-193a-3p in the EGFR-related signaling pathway [37]. Using human non-small cell lung cancer samples and cell lines, they showed that miR-193a-3p and miR-193a-5p are under-expressed in non-small cell lung cancer, particularly in metastatic tumors of lung cancer, and that the overexpression of miR-193a-3p and miR-193a-5p leads to the inhibition of lung cancer metastasis in vitro and in vivo. The inhibitory effect of miR-193a-3p and miR-193a-5p on metastasis may be due to the down-regulation of the ERBB4/PIK3R2/mTOR/S6 K2 signaling pathway through direct targeting of *ERBB4* and *S6 K2* by miR-193a-3p, and *PIK3R3* and mTOR by miR-193a-5p [37]. Their findings were supported by another recent study that confirmed that miR-193a-3p directly targets ERBB4 in lung cancer [47]. Furthermore, surprisingly, a more recent report has revealed that miR-193a-3p directly targets *KRAS* and inhibits tumor growth and metastasis in an ex vivo and in vivo lung cancer model [48]. These lines of evidence, together with our results, support a notion that miR-193a-3p acts as a negative regulator for the EGFR/ERBB-related pathways in various cancers, although the full mechanisms underlying the miR-193a-3p-EGFR/ERBB regulatory network in colorectal cancer require further elucidation.

Another question is how miR-193a-3p is initially down-regulated in cancer cells. Our results that miR-193a-3p expression is modestly, but significantly, decreased by the overexpression of BRAF mutant protein in non-*BRAF*-mutant cell lines (Fig. 3d) suggest the possibility that miR-193a-3p may directly regulate the EGFR-related pathway and also be regulated, at least in part, directly by BRAF or other downstream EGFR-related pathways within a feedback loop. However, both BRAF and MEK inhibition did not significantly affect miR-193a-3p expression in cells with already activated BRAF or KRAS. The possible mechanism of miR-193a-3p down-regulation via BRAF is likely more complicated. Furthermore, the luciferase assay has shown that the only modest decrease in miR-193a-3p expression induced by transient BRAF overexpression (by about 20%), which is a minor difference compared with the 2–3-fold difference observed between patients with *BRAF* mutations and those without *KRAS/BRAF* mutations (Figs. 1 and 2), did not lead to the recovery of expression of target genes (Additional file 4: Figure S3). Together, our results suggest that mutant BRAF may partly affect miR-193a-3p expression, but is unlikely to be a main direct cause of alterations in miR-193a-3p and its target gene expressions that are involved in tumorigenesis of colorectal cancer. Several earlier reports have raised another explanation for the mechanism for the down-regulation of miR-193a-3p. miR-193a is located in 17q11.2, and this miRNA and its promoter region are located within CpG islands. The promoter DNA methylation of miR-193a has been implicated in oral cancer [40], acute myeloid leukemia [41], and non-small cell lung cancer [42]. The precise mechanism underlying this miRNA down-regulation, particularly in *BRAF*-mutated colorectal cancer, should be further elucidated in future studies.

In addition, a clinically important novel finding of this study was that miR-193a-3p expression status was associated with the clinical outcome of colorectal cancer patients treated with anti-EGFR therapy. The miR-193a-3p expression status did not correlate with OS or PFS for cytotoxic drug therapies, but low expression status was associated with a worse PFS for anti-EGFR therapies among all patients analyzed (Fig. 4). Even when only patients without *KRAS*/*BRAF* mutations were analyzed, a similar trend was observed (Fig. 4), although it should be noted that the difference did not reach significance due to the limited number of patients. Our study was a retrospective setting, and the sample size used in the survival analysis was relatively small to draw a robust conclusion on the effect of miR-193a-3p dysregulation on the sensitivity to anti-EGFR therapy. Therefore, the association between miR-193a-3p and the outcomes of anti-EGFR therapy should be confirmed in larger, prospective studies. Nevertheless, the possible correlation between miR-193a-3p expression and the clinical outcome from anti-EGFR therapy in patients with colorectal cancer further support a role of miR-193a-3p in the EGFR-related signaling pathway.

## Conclusion

In conclusion, we present novel evidence that miR-193a-3p is down-regulated in colorectal cancer, specifically *BRAF*-mutant colorectal cancer, and that miR-193a-3p acts as a tumor suppressor in colorectal cancer. Our study additionally describes a potential role of this miRNA as a novel biomarker for predicting who can benefit from anti-EGFR therapy. Our findings provide a rationale for conducting further studies on the precise molecular mechanisms underlying miR-193a-3p regulation and its contribution to colorectal carcinogenesis, which could lead to identifying new molecular targets for therapy and thus lead to developing the new therapeutic strategies for patients with colorectal cancer and possibly other malignant diseases.

## Additional files

Additional file 1: Table S1. Clinical characteristics of patients with colorectal cancer in this study. **Table S2.** Tumor response of patients with colorectal cancer who received anti-EGFR therapy based upon the miR-193a-3p expression status. **Table S3**. Tumor response of patients with *KRAS/BRAF*-wild-type colorectal cancer who received anti-EGFR therapy based upon the miR-193a-3p expression status. (DOCX 27 kb)

Additional file 2: Figure S1. Correlations between the microarray results and the qPCR results in a screening set (*n* = 30). The signal intensities obtained by microarray analysis were well correlated with the expression results determined by qPCR for **a** miR-193a-3p and **b** miR-16. Pearson’s correlation coefficient was presented. (PPTX 42 kb)

Additional file 3: Figure S2. Influence of a treatment with a BRAF inhibitor and a MEK inhibitor on miR-193a-3p expression. miR-193a-3p expression was measured in a BRAF-mutant cell line RKO (left panel) and a KRAS-mutant cell line HCT116 (right panel) treated with a BRAF inhibitor dabrafenib (D-5699, LC laboratories, MA, USA) and a MEK inhibitor trametinib (16,292, Cayman Chemical Company, MI, USA) in multiple timepoints and multiple doses. Data are obtained from two independent experiments. (PPTX 138 kb)

Additional file 4: Figure S3. Influence of mutant BRAF overexpression on target sequences of miR-193a-3p. A psiCHECK-2 vector that has a luciferase sequence with target sequences of miR-193a-3p (miCheck miRNA biosensor clone, Promega, WI, USA), and either the BRAF V600E overexpression vector (pBRAF) or control vector (pEGFP) were co-transfected into KRAS/BRAF-wild SW48 cells. Luciferase activity was measured 30 h after the transfection. Data are represented as mean + −SE from three independent experiments. (PPTX 76 kb)

Additional file 5: Figure S4. Kaplan–Meier curves for overall survival (OS) and progression-free survival (PFS) of the patients with colorectal cancer who received the first-line chemotherapy. **a** OS and **b** PFS for the first-line chemotherapy according to the KRAS/BRAF mutational status (*n* = 99). The hazard ratio (HR) of OS for the KRAS-mutant group and the BRAF-mutant group against the KRAS/BRAF-wild-type group were 1.15 (95% CI, 0.66 to 1.95, *P* = 0.62) and 3.44 (1.44 to 7.38, *P* < 0.01). The HR of PFS and OS for the KRAS-mutant group and the BRAF-mutant group against the KRAS/BRAF-wild-type group were 0.97 (0.60 to 1.58, *P* = 0.93) and 0.83 (0.34 to 1.80, *P* = 0.67). **c** OS and **d** PFS for the first-line chemotherapy according to the miR-193a-3p expression group (n = 99). The HR of OS and PFS for the low expression group against the high expression group was 0.79 (0.48 to 1.30, *P* = 0.35) and 1.11 (0.70 to 1.76, P = 0.67). A log-rank test was used to analyze the statistical differences in survival. (PPTX 59 kb)

## Abbreviations

CIMP: CpG island methylator phenotype
FFPE: Formalin-fixed paraffin-embedded
miR: miRNA
MSI: Microsatellite instability
NCCH: National Cancer Center Hospital
OS: Overall survival
PFS: Progression-free survival
qRT-PCR: Real-time RT-PCR
RR: Response rate
TUH: Tohoku University Hospital
