# Supplementary material for: microRNA-193a-3p is specifically down-regulated and acts as a tumor suppressor in BRAF-mutated colorectal cancer
Source: BMC Cancer. 2017 Nov 7;17:723. doi: 10.1186/s12885-017-3739-x (PMC5678600; doi:10.1186/s12885-017-3739-x)
Supplement: Supplementary file 1 — Clinical characteristics of patients with colorectal cancer in this study. Table S2. Tumor response of patients with colorectal cancer who received anti-EGFR therapy based upon the miR-193a-3p expression status. Table S3. Tumor response of patients with KRAS/BRAF-wild-type colorectal cancer who received anti-EGFR therapy based upon the miR-193a-3p expression status. (DOCX 27 kb) [file 12885_2017_3739_MOESM1_ESM.docx]

**Table S1**. Clinical characteristics of patients with colorectal cancer in this study.

| Characteristic |  | Tohoku University Hospital (n = 255) | | | | National Cancer Center Hospital  (n = 59) | | | |  |
| --- | --- | --- | --- | --- | --- | --- | --- | --- | --- | --- |
|  |  | n |  | % |  | n |  | % |  | *P* |
| Age | median | 64 |  |  |  | 62 |  |  |  | 0.08^a^ |
|  | range | 16 - 92 |  |  |  | 29 - 83 |  |  |  |  |
| Gender | men | 157 |  | 61 |  | 43 |  | 73 |  | 0.13^b^ |
|  | women | 98 |  | 38 |  | 16 |  | 27 |  |  |
| Stage | I | 2 |  | 1 |  | 1 |  | 2 |  | 0.04^c^ |
|  | II | 25 |  | 10 |  | 4 |  | 7 |  |  |
|  | III | 88 |  | 35 |  | 15 |  | 25 |  |  |
|  | IV | 98 |  | 38 |  | 39 |  | 66 |  |  |
|  | unknown | 42 |  | 16 |  | 0 |  | 0 |  |  |
| Histology | pap | 2 |  | 1 |  | 0 |  | 0 |  | 0.35^c^ |
|  | tub | 174 |  | 68 |  | 55 |  | 93 |  |  |
|  | por | 21 |  | 8 |  | 3 |  | 5 |  |  |
|  | muc | 13 |  | 5 |  | 1 |  | 2 |  |  |
|  | sig | 1 |  | 0 |  | 0 |  | 0 |  |  |
|  | unknown | 44 |  | 17 |  | 0 |  | 0 |  |  |
| Location | proximal | 89 |  | 35 |  | 13 |  | 22 |  | 0.006^b^ |
|  | distal | 126 |  | 49 |  | 46 |  | 78 |  |  |
|  | unknown | 40 |  | 16 |  | 0 |  | 0 |  |  |
| *KRAS/BRAF* status | wt/wt | 143 |  | 56 |  | 53 |  | 89 |  | < 0.0001^c^ |
|  | mt/wt | 91 |  | 35 |  | 4 |  | 7 |  |  |
|  | wt/mt | 19 |  | 8 |  | 2 |  | 3 |  |  |
|  | mt/mt | 2 |  | 1 |  | 0 |  | 0 |  |  |
| ^a^Mann-Whitney U test, ^b^Fisher's exact test, ^c^chi-square tests were used for the comparison of categorical variables between the two cohorts. | | | | | | | | | | |
| Abbreviations: wt, wild type; mt, mutant type; pap, papillary adenocarcinoma; tub, tubular adenocarcinoma; por, poorly differentiated adenocarcinoma; muc, mucinous adenocarcinoma; sig, signet-ring cell carcinoma. | | | | | | | | | | |

**Table S2**. Tumor response of patients with colorectal cancer who received anti-EGFR therapy based upon the miR-193a-3p expression status.

|  | High miR-193a-3p  expression (n = 23) | | | | Low miR-193a-3p  expression (n = 22) | | | |  |
| --- | --- | --- | --- | --- | --- | --- | --- | --- | --- |
| Response | n |  | % |  | n |  | % |  | *P* |
| CR | 0 |  | 0 |  | 0 |  | 0 |  |  |
| PR | 8 |  | 42 |  | 5 |  | 24 |  |  |
| SD | 7 |  | 37 |  | 9 |  | 43 |  |  |
| PD | 4 |  | 21 |  | 7 |  | 33 |  |  |
| NE | 4 |  |  |  | 1 |  |  |  |  |
|  |  |  |  |  |  |  |  |  |  |
| RR |  |  | 42 |  |  |  | 24 |  | 0.31^a^ |
| DCR |  |  | 79 |  |  |  | 67 |  | 0.49^a^ |
| ^a^Fisher's exact test was used for the comparison of RR and DCR, between the high expression group and low expression group. | | | | | | | | | |
| Abbreviations: CR, complete response; PR, partial response; SD, stable disease;  PD, progressive disease; NE, Not evaluable; RR, response rate; DCR, disease control rate. | | | | | | | | | |

**Table S3**. Tumor response of patients with *KRAS/BRAF-wild-type* colorectal cancer who received anti-EGFR therapy based upon the miR-193a-3p expression status.

|  | High miR-193a-3p  expression (n = 20) | | | | Low miR-193a-3p  expression (n = 14) | | | |  |
| --- | --- | --- | --- | --- | --- | --- | --- | --- | --- |
| Response | n |  | % |  | n |  | % |  | *P* |
| CR | 0 |  | 0 |  | 0 |  | 0 |  |  |
| PR | 8 |  | 47 |  | 4 |  | 31 |  |  |
| SD | 6 |  | 35 |  | 5 |  | 38 |  |  |
| PD | 3 |  | 17 |  | 4 |  | 31 |  |  |
| NE | 3 |  |  |  | 1 |  |  |  |  |
|  |  |  |  |  |  |  |  |  |  |
| RR |  |  | 47 |  |  |  | 31 |  | 0.47^a^ |
| DCR |  |  | 82 |  |  |  | 69 |  | 0.67^a^ |
| ^a^Fisher's exact test was used for the comparison of RR DCR between high expression group and low expression group. | | | | | | | | | |
| Abbreviations: CR, complete response; PR, partial response; SD, stable disease; PD, progressive disease; NE, Not evaluable; RR, response rate; DCR, disease control rate. | | | | | | | | | |
