# Supplementary material for: microRNA-193a-3p is specifically down-regulated and acts as a tumor suppressor in BRAF-mutated colorectal cancer
Source: BMC Cancer. 2017 Nov 7;17:723. doi: 10.1186/s12885-017-3739-x (PMC5678600; doi:10.1186/s12885-017-3739-x)
Supplement: Supplementary file 2 — Correlations between the microarray results and the qPCR results in a screening set (n = 30). The signal intensities obtained by microarray analysis were well correlated with the expression results determined by qPCR for a miR-193a-3p and b miR-16. Pearson’s correlation coefficient was presented. (PPTX 42 kb) [file 12885_2017_3739_MOESM2_ESM.pptx]

## Slide 1
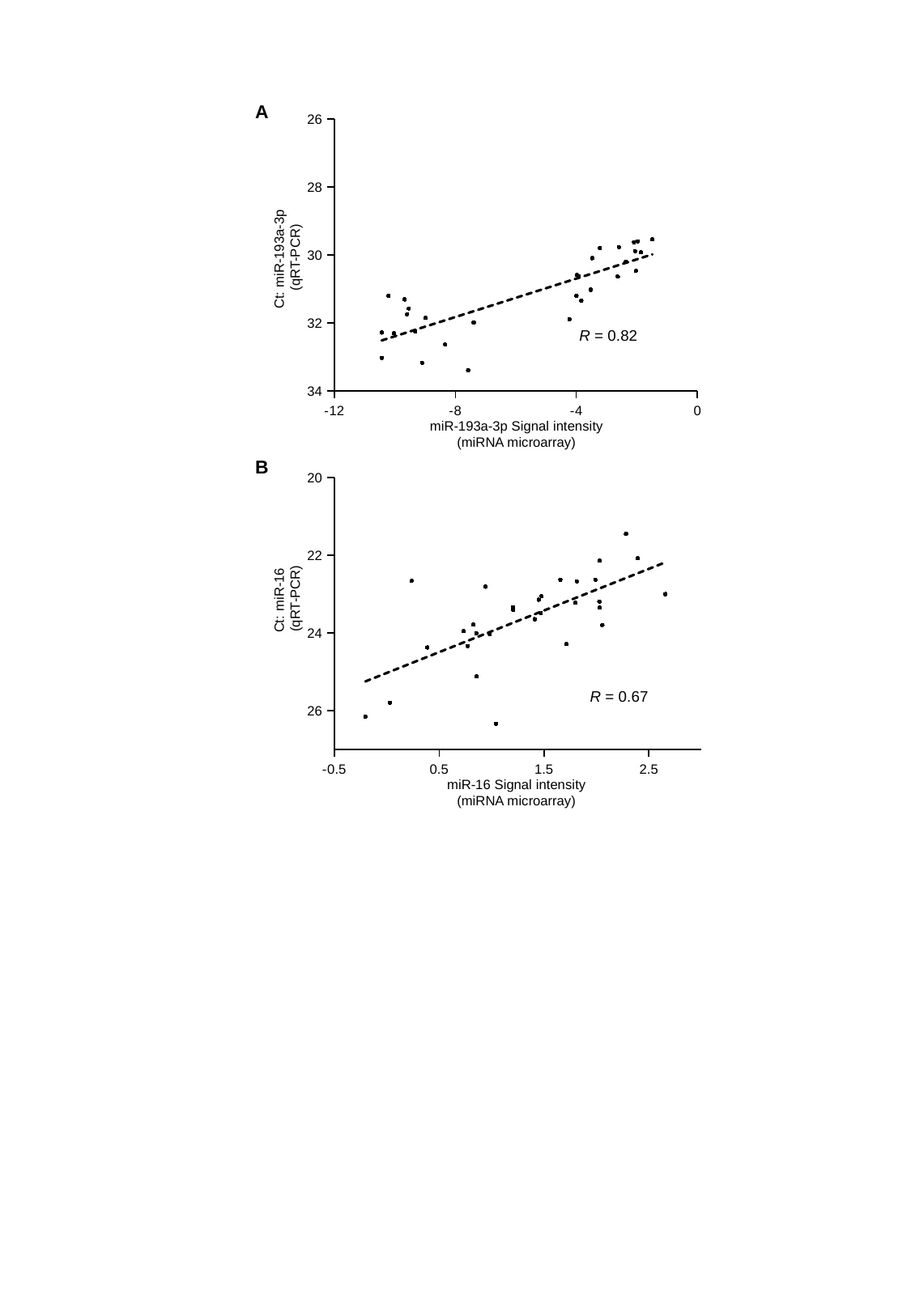

A
### Chart
| Category | |
|---|---|Ct: miR-193a-3p
 (qRT-PCR)
R = 0.82
miR-193a-3p Signal intensity
(miRNA microarray)
B
### Chart
| Category | | |
|---|---|---|Ct: miR-16
 (qRT-PCR)
R = 0.67
miR-16 Signal intensity
(miRNA microarray)
