# Supplementary material for: microRNA-193a-3p is specifically down-regulated and acts as a tumor suppressor in BRAF-mutated colorectal cancer
Source: BMC Cancer. 2017 Nov 7;17:723. doi: 10.1186/s12885-017-3739-x (PMC5678600; doi:10.1186/s12885-017-3739-x)
Supplement: Supplementary file 3 — Influence of a treatment with a BRAF inhibitor and a MEK inhibitor on miR-193a-3p expression. miR-193a-3p expression was measured in a BRAF-mutant cell line RKO (left panel) and a KRAS-mutant cell line HCT116 (right panel) treated with a BRAF inhibitor dabrafenib (D-5699, LC laboratories, MA, USA) and a MEK inhibitor trametinib (16,292, Cayman Chemical Company, MI, USA) in multiple timepoints and multiple doses. Data are obtained from two independent experiments. (PPTX 138 kb) [file 12885_2017_3739_MOESM3_ESM.pptx]

## Slide 1
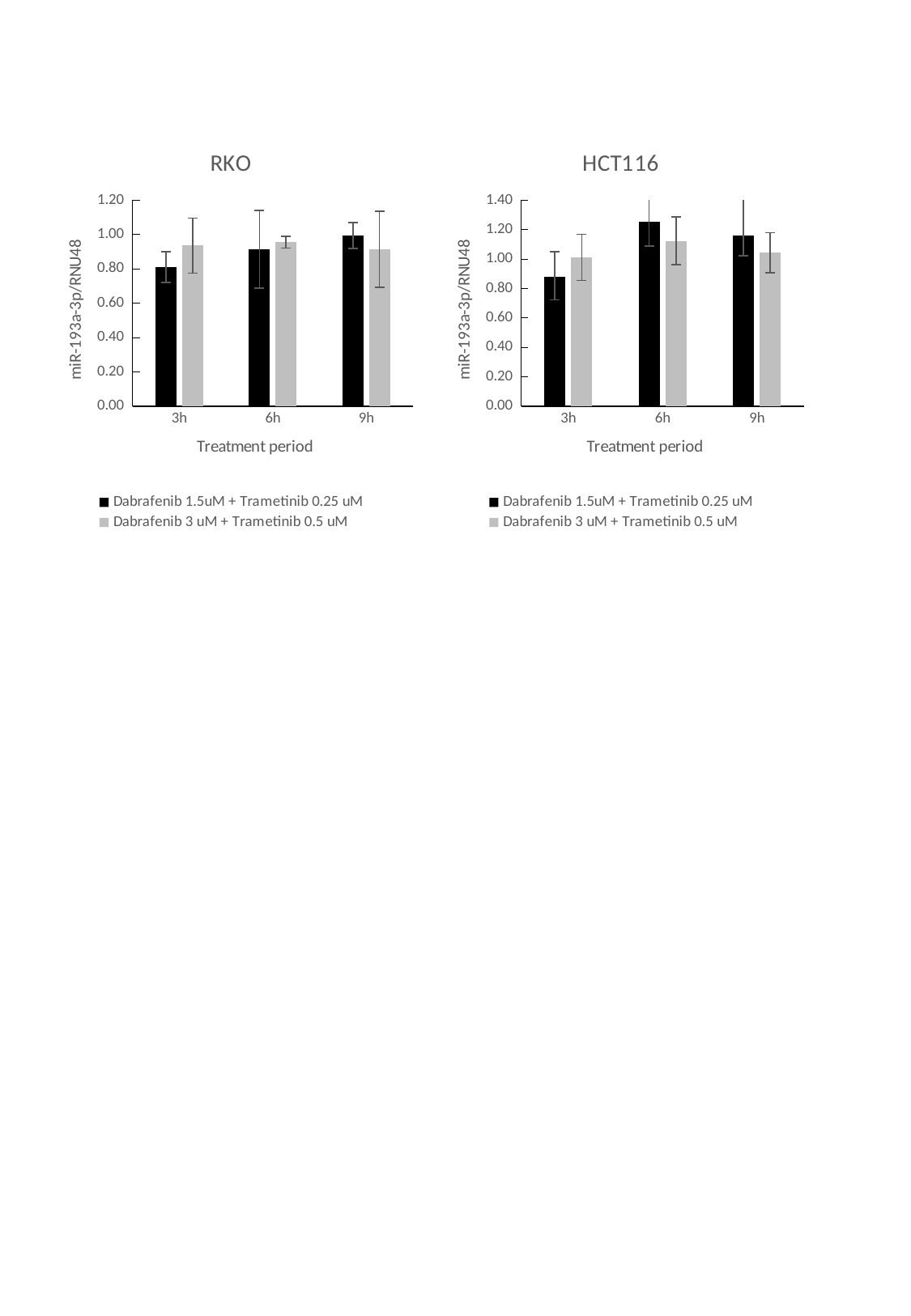

### Chart: RKO
| Category | Dabrafenib 1.5uM + Trametinib 0.25 uM | Dabrafenib 3 uM + Trametinib 0.5 uM |
|---|---|---|
| 3h | 0.8105187216640779 | 0.9363021537306443 |
| 6h | 0.9141740281549042 | 0.9559124945175423 |
| 9h | 0.9940038887752514 | 0.9145472141814073 |
### Chart: HCT116
| Category | Dabrafenib 1.5uM + Trametinib 0.25 uM | Dabrafenib 3 uM + Trametinib 0.5 uM |
|---|---|---|
| 3h | 0.8811180441995637 | 1.0121118552150334 |
| 6h | 1.2519556748856435 | 1.124092856219884 |
| 9h | 1.1572461481618102 | 1.0436972375287086 |
