# Supplementary figures and images for: microRNA-193a-3p is specifically down-regulated and acts as a tumor suppressor in BRAF-mutated colorectal cancer
Source: BMC Cancer. 2017 Nov 7;17:723. doi: 10.1186/s12885-017-3739-x (PMC5678600; doi:10.1186/s12885-017-3739-x)

## Slide 1
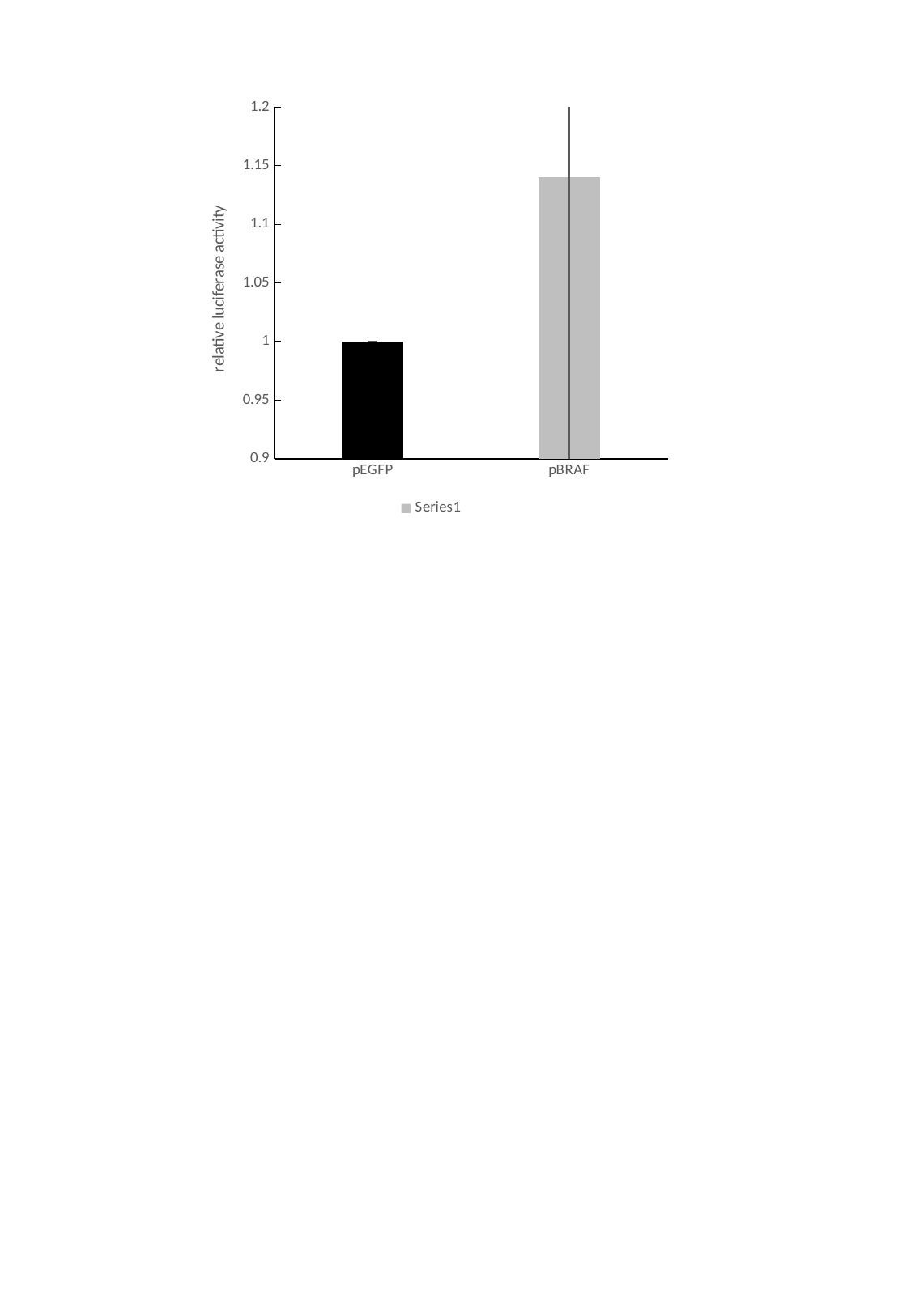

### Chart
| Category | |
|---|---|
| pEGFP | 1.0 |
| pBRAF | 1.1403964984569452 |

Supplement: Supplementary file 4 — Influence of mutant BRAF overexpression on target sequences of miR-193a-3p. A psiCHECK-2 vector that has a luciferase sequence with target sequences of miR-193a-3p (miCheck miRNA biosensor clone, Promega, WI, USA), and either the BRAF V600E overexpression vector (pBRAF) or control vector (pEGFP) were co-transfected into KRAS/BRAF-wild SW48 cells. Luciferase activity was measured 30 h after the transfection. Data are represented as mean + −SE from three independent experiments. (PPTX 76 kb) [file 12885_2017_3739_MOESM4_ESM.pptx]
