# Supplementary material for: microRNA-193a-3p is specifically down-regulated and acts as a tumor suppressor in BRAF-mutated colorectal cancer
Source: BMC Cancer. 2017 Nov 7;17:723. doi: 10.1186/s12885-017-3739-x (PMC5678600; doi:10.1186/s12885-017-3739-x)
Supplement: Supplementary file 5 — Kaplan–Meier curves for overall survival (OS) and progression-free survival (PFS) of the patients with colorectal cancer who received the first-line chemotherapy. a OS and b PFS for the first-line chemotherapy according to the KRAS/BRAF mutational status (n = 99). The hazard ratio (HR) of OS for the KRAS-mutant group and the BRAF-mutant group against the KRAS/BRAF-wild-type group were 1.15 (95% CI, 0.66 to 1.95, P = 0.62) and 3.44 (1.44 to 7.38, P < 0.01). The HR of PFS and OS for the KRAS-mutant group and the BRAF-mutant group against the KRAS/BRAF-wild-type group were 0.97 (0.60 to 1.58, P = 0.93) and 0.83 (0.34 to 1.80, P = 0.67). c OS and d PFS for the first-line chemotherapy according to the miR-193a-3p expression group (n = 99). The HR of OS and PFS for the low expression group against the high expression group was 0.79 (0.48 to 1.30, P = 0.35) and 1.11 (0.70 to 1.76, P = 0.67). A log-rank test was used to analyze the statistical differences in survival. (PPTX 59 kb) [file 12885_2017_3739_MOESM5_ESM.pptx]

## Slide 1
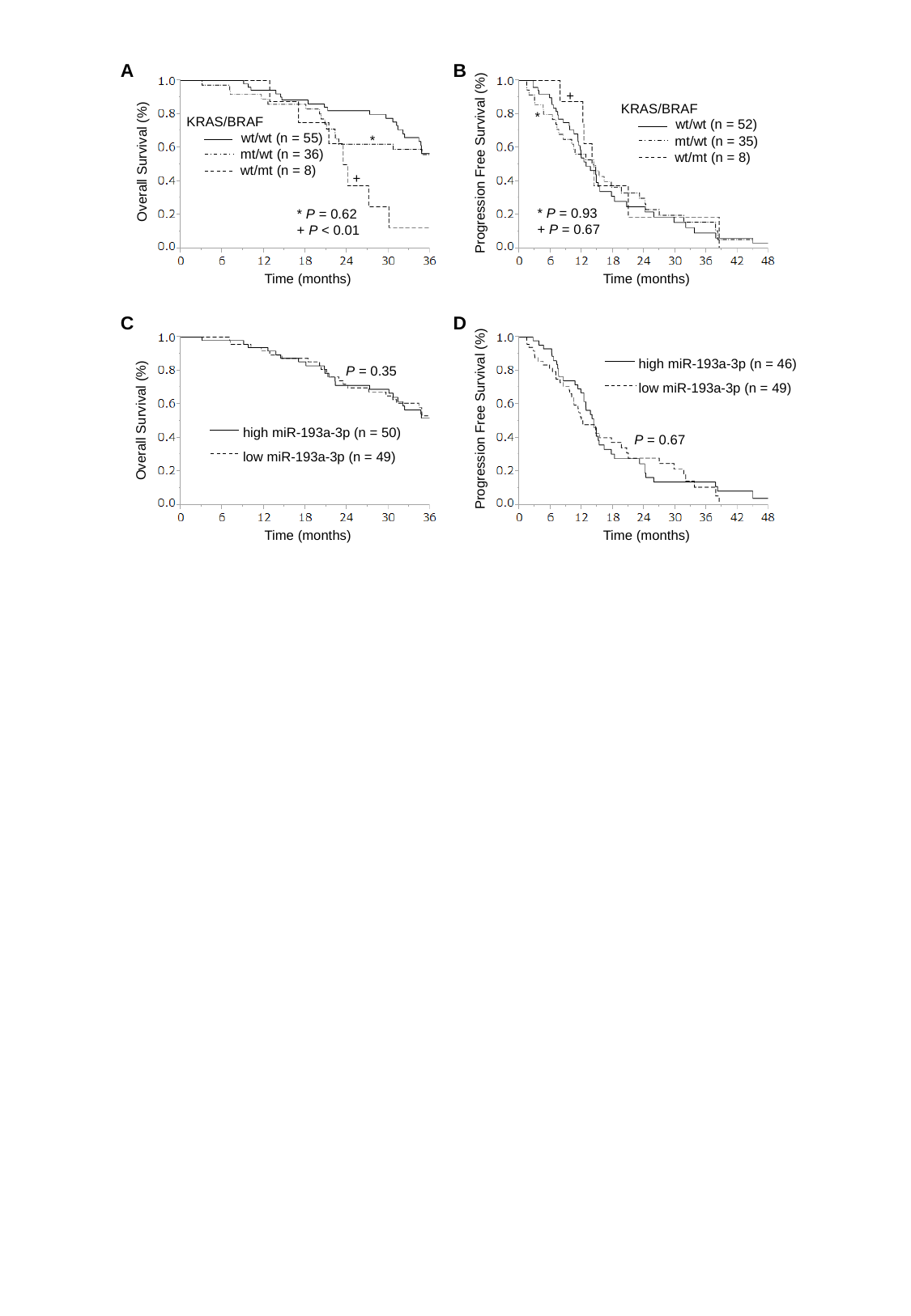

A
B
+
KRAS/BRAF
　 wt/wt (n = 52)
 mt/wt (n = 35)
 wt/mt (n = 8)
*
KRAS/BRAF
　 wt/wt (n = 55)
 mt/wt (n = 36)
 wt/mt (n = 8)
*
Overall Survival (%)
Progression Free Survival (%)
+
* P = 0.93
+ P = 0.67
* P = 0.62
+ P < 0.01
Time (months)
Time (months)
C
D
high miR-193a-3p (n = 46)
low miR-193a-3p (n = 49)
P = 0.35
Progression Free Survival (%)
Overall Survival (%)
high miR-193a-3p (n = 50)
low miR-193a-3p (n = 49)
P = 0.67
Time (months)
Time (months)
